# Collage-based narratives of mothers of third culture kids: the in-between space of transnational families in the UAE

**DOI:** 10.3389/fsoc.2024.1497479

**Published:** 2024-11-13

**Authors:** Anna Marie Dillon, Tabassim Ali

**Affiliations:** ^1^Emirates College for Advanced Education, Abu Dhabi, United Arab Emirates; ^2^Zayed University, Abu Dhabi, United Arab Emirates

**Keywords:** expatriates, motherhood, third culture kids, identity, narrative, visual research method, families, transnational

## Abstract

This study delves into the lives of transnational families in the UAE, exploring their complex identities using a visual research approach known as the Collage Life Elicitation Technique (CLET). Both the topic and the methodology are framed within a theoretical lens of *Verstehen* (understanding) with a view to further exploring the lives of transnational families in the context of a pragmatic phenomenology and within the setting of the UAE as a rentier state. The lives of families such as these are characterized by a cross-cultural lifestyle, high mobility and expected repatriation. This paper presents narratives of eight transnational families in the UAE as expressed by the mother of each family unit, and seeks to expand on how these mothers view the identity of their families as expatriates living in the UAE. Findings indicate that these families navigate an intricate world where they are neither entirely of their host country nor of their passport country. The family unit plays a pivotal role in these families’ lives, acting as a bridge between their host culture and their home culture, serving as a nucleus for their evolving identities.

## Introduction

This paper seeks to explore the narratives of transnational families in the United Arab Emirates (UAE), using the exploratory tool of the Collage Life Elicitation Technique (CLET; Van Schalkwyk, 2010; Lijadi and Van Schalkwyk, 2014). Both the topic and the methodology are framed within a theoretical lens of *Verstehen* (understanding) with a view to further exploring the lives of transnational families in the context of a pragmatic phenomenology and within the setting of the UAE as a rentier state.

The study aims to gain a better understanding of the ways in which transnational families view their lived experiences as expatriates in the UAE. By offering participants a unique opportunity to explore their families’ lives by narrating their experiences both verbally and visually, it was hoped to capture the richness of these narratives as told by the mothers of these families, thereby providing a snapshot of their lived experiences at that time. The term TCK is a commonly used term to denote children living in a host culture other than their passport culture during their developmental years (Dillon and Ali, 2019). We refer to transnational families in this study as we are interested in the experiences of families, as related by mothers, rather than individuals. Transnational families are exposed to multiple cultural settings as part of the international diaspora of globally mobile expatriates. The lives of families such as these are characterized by a cross-cultural lifestyle, high mobility and expected repatriation (de Waal and Born, 2021). This paper presents narratives of eight transnational families in the UAE as expressed by the mother of each family unit, and seeks to expand on how these mothers view the identity of their families as expatriates living in the UAE.

According to Lijadi and van Schalkwyk “The CLET has great potential as a visual research method in different contexts and for various populations” (Lijadi and Van Schalkwyk, 2014, p. 4). The authors chose this technique because of the unique affordances it presents. CLET was developed to explore life story “remembering” in cross-cultural settings where the combination of non-verbal and verbal strategies provide a meaningful way for the researchers and participants to co-construct knowledge. The utilization of the CLET can provide us with ways to elicit unstructured and spontaneous versions of the individual’s perspective of this topic. This autobiographical approach can stimulate the recollection of her/his self-defining memories in this area and therefore produce rich narratives. They were asked to select images which represent something important or memorable about language and identity in their family’s lives. This process helped us to answer the research question—what are the experiences of transnational families in the UAE, from the perspectives of mothers?

Participants in this study were asked to create collages that represented important their families’ lives, while keeping in mind their lives as families living away from their passport countries. In this way, the mothers of TCKs were afforded an opportunity to express their families’ lived experiences as transnational families in the UAE.

### Third culture families in the UAE and beyond

The UAE is the host of a very distinctive demographic landscape when comparing the number of citizens and non-citizens, as noted by Hopkyns when she says “the UAE lingustic and cultural ecology is incredibly diverse” (Hopkyns, 2023, p. 283). It is a rentier state, with its economy strongly driven by selling oil, one of its main natural resources (Errichiello and Nyhagen, 2021; Dillon and Ali, 2019), although the economy has diversified considerably in the last number of years. Many rentier states have a high proportion of expatriate workers, and the UAE is no different. Similarly to cities in the Asia-Pacific region such as Singapore and Hong Kong, global cities in the Gulf Cooperation Council attract both low-and high-skilled migrant workers (Benuyenah and Mustafa, 2024). In fact, in the emirate of Abu Dhabi, Emirati national citizens made up almost 20% of the population in 2016, while in the commercial center emirate of Dubai less than 10% of the population was Emirati in 2017 (Gallagher, 2019). The UAE is an example of a particular context where the “host culture” (Emiratis) is a minority (Baycar, 2023; Hopkyns, 2023).

Transnational families in the UAE present in a variety of ways in nuclear middle-class families, just as they do in many countries around the world. In some cases, both parents are working full-time, with their children either attending school or nursery, or cared for by a nanny at home. In other cases, the male spouse is the only earner in the household, and in other (rarer) cases the female spouse might be the only earner. The types of well-paid positions jobs traditionally held in the oil and gas sector meant that in the past, males had more opportunities for better paid positions, even where the female spouse would have been previously employed in their home country (Cangià, 2018). However, with demographics within the labor market constantly changing (De Bel-Air, 2018), it is unhelpful to make generalizations other than to note that the female spouse tends to take responsibility for the traditionally gendered tasks in the home, even in cases where she is a mother and working full-time, as noted by Dickson (2019). This gendered role taps into the phenomenon of the “trailing spouse” (Cangià, 2018, p. 8), which involves the wife accompanying the husband in mobility, seeing an intensification in household chores, or where there is not an intensification in household chores, the employment of a nanny or live-in helper, something that usually would not have been financially possible in the home country. The role of trailing spouse can lead to more traditionally gendered roles in transnational families, adding to the already existing disproportionate burden on women as gatekeepers of the domestic sphere in all its forms. It is well known that mothers tend to play more dominant roles in caregiving within the family, and tend to spend more time with their children even if they are working full-time (Pancsofar et al., 2019), although Dillon et al. (2024) indicates that fathers are taking more of a role in child-rearing in the context of the UAE. These realities are important to acknowledge as the phenomenon of transnational families in the UAE is explored throughout this paper from the perspective of mothers, whether working at home or outside the home.

The lives of expatriate families tend to be characterized by mobility (either theirs or that of others in their lives). This mobility can lead to an “almost perpetual state of psychological transition” (Gilbert, 2008, p. 94). A participant interviewed by Errichiello and Nyhagen (2021) referred to Dubai as a transit lounge in and of itself. The mobility can lead to feelings of opportunity, or feelings of loss, a sense of belonging everywhere, or a sense of belonging nowhere. Gilbert’s study focuses on feelings of loss, and found that many losses could be categorized in relation to persons, places, pets, possessions, as well as existential losses. Gillies (1998) highlights some of the affordances and challenges for expatriate families. Some of the affordances identified include an expanded world view, international perspective and exposure to myriad cultures, as well as the potential to become competent in multiple languages. Some challenges highlighted include loneliness, difficulties in maintaining friendships in the host country and in the home country, and a lack of knowledge about popular culture either in the host or home culture. Some of these losses may be hidden, even if TCKs return to their passport countries – being unfamiliar with the dominant culture while perhaps sounding similar to others within their home countries may lead to being a sort of hidden immigrant, with a hidden diversity that may not be apparent to others (de Waal and Born, 2021; Pollock and Van Reken, 2017). Parents within transnational families may express concern about the potential disruption caused by their globally mobile lifestyle to their children’s social networks and identities, but often the focus of research has been on the TCKs within these family units, and not the family as a whole. Recently, Khonder’s chapter (Khonder, 2022) exploring the identities of Bangladeshi adult TCKs living in the UAE found ambivalence to be a key feature and indeed strength of their narratives, rather than the more traditional view of cultural homelessness attributed to TCKs.

This paper is underpinned by the idea of Thirdness, both in terms of conceptualizing TCK mothers’ sense of their families’ selves, and in terms of the autobiographical and interpretive methodological approach used to draw out this sense of self. It is viewed through the theoretical lens of *Verstehen*, which seeks to understand meaning from the individual’s perspective.

### Thirdness—cultures of TCKs

Classic pragmatic philosophers such as Peirce consider that there are three fundamental phenomenological categories—Firstness, Secondness, and Thirdness (Raggatt, 2010). When these categories interact with each other, they form a triad. A TCK, a TCI (Third Culture Individual) or a transnational family can be viewed as triadic. In each TCK we can find “a first bound together with a second by the mediation of a third” (Raggatt, 2010, p. 401). This relates to the classic description of a TCK (Pollock and van Reken, 2017), which sees the first culture (Firstness) referring to the passport country, the second culture (Secondness) to the host culture, and the third culture (Thirdness) as the trans-cultural intervening space or “interstitial culture” (Lijadi and Van Schalkwyk, 2014, p. 1). Given that the first and second cultures are tangible social structures, while the third culture is less tangible and much more transient and difficult to define, the idea of Thirdness is challenging to conceptualize. The meaning of this “Third” intervening space becomes dependent on how the Thirdness is mediated through the Firstness and Secondness. Bhabha’s conception of these in-between spaces as individuals are “living on the borderlines of the present” (Bhandari, 2020, p. 81) in a continually shifting condition. Just as the prefix “post” in jargons such as postcolonialism and postmodernism do not signify sequentiality, the “third” in the jargons of TCK or Thirdness do not indicate succession. Rather, it is an intervening space, including aspects of pre-existing cultural practices, developing cultural practices, and in addition to an amalgamation of these the space mediates a newness that can be “creative, fertile and unstable” (Bhandari, 2020, p. 82). As explained by Bhabha “it renews the past, refiguring it as a contingent “in-between” space, that innovates and interrupts the performance of the present” (Bhabha, 1994, p. 7).

As the Thirdness, or third culture, is an elusive concept, and can involve subtractive feelings such as feeling like not belonging in one place or another, as well as additive feelings such as feeling like belonging everywhere, it is important to explore the sense of self. Raggatt (2010) highlights the contributions made by Hermans and Bakhtin in defining the dialogical self. He sees it as a “dialogical multiplicity of several I-positions that have separate stories to tell” (Raggatt, 2010, p. 405). The “I” is capable of shifting from one position to another. The self in this view is continually evolving and has separate stories to tell. This draws on (Woźniak, 2018) work which accounted for a number of Me’s, such as empirical, spiritual and social (Raggatt, 2010). The TCI can be seen as a tangible representation of the dialogical self with several I-positions and several versions of “Me.” Indeed, the interstitial space is comprised of not simply an amalgamation of the first and second culture, but a way of life shared by others who also grew up or have spent considerable time between worlds (Pollock and van Reken, 2017). These others may include friends, acquaintances, teachers or family members.

### Thirdness as methodological approach—collage life elicitation technique

We can also apply Thirdness to the methodological approach taken in this paper, in the sense outlined by Raggatt’s methodological approach to autobiographical case studies (2010). By using the CLET, the self is “constituted through language and symbolic behavior” (Raggatt, 2010, p. 401). The language is found through the interview and naturalistic conversations, while the symbolism is found through the images chosen by participants and the creation of a collage representing their family’s self. Van Schalkwyk developed the CLET as a “unique method geared toward a deeper understanding of the symbolism informing the narrative meaning-making process” (Van Schalkwyk, 2010, p. 677). She notes that providing participants with an opportunity to narrate their life stories using images facilitates the process of “making sense of past selves, past events and past circumstances within the context of social categories and in social interaction” (Van Schalkwyk, 2010, p. 677).

This semiotic model, a visual non-verbal narrative accompanied by a verbal narrative, allows participants to engage in constructing a dialogical self theory of their families, and affords participants the opportunity to represent that self by using a multiplicity of signs (Raggatt, 2010). Raggatt (2010) holds that the self can be best constructed within this semiotic framework for mediation. Using this type of visual research method, which stimulates remembering to elicit rich narratives, might lessen any “cultural inhibition toward self-disclosure” (Lijadi and Van Schalkwyk, 2014, p. 4). It also acknowledges the power of social action and provides opportunities for participants to co-create experiences and stories about their world and take co-ownership for their “representations of reality as perceived and experienced” (Lijadi and Van Schalkwyk, 2014, p. 4). The CLET is underpinned by the theoretical framework proposed by the narrative and social constructionist approaches to knowledge construction. Within the social constructionist approach, if the definition of language is broadened to include non-verbal or visual languages, artifacts can add meaning to the social process of co-constructing our thoughts, intentions, experiences, memories, creativity, and co-action.

### Theoretical lens

The theoretical lens adopted is that of *Verstehen*, a concept acknowledged within philosophy and interpretive sociology. *Verstehen* (literally translated from German to English as “understanding”) refers to understanding meaning from the individual’s perspective. It involves empathizing or participating with another individual, and acknowledging that individuals create actions within their context by organizing their own understandings and attributing meaning to those actions. The *Stanford Encyclopedia of Philosophy* states that “It is a reflective mode of inquiry that provides the framework for more specific explanations, whether causal or rational” (Stanford Encyclopedia of Philosophy, n.d.).

The concept of *Verstehen* draws on the work of Weber and Dilthey (Rickman, 1979) and is concerned with the theory and practice of interpretation. Dilthey was particularly interested in the “life-context” of understanding, which holds resonance for the present study. As all of the participants in this study are interlocutors during the data collection and contributing to each other’s thoughts, *Verstehen* comes from the discussions that ensue as they empathize, participate and reflect on action and in action with each other. Dilthey held that that understanding comes from individual worldviews, interpretations, and a shared world (Palmer, 1969), which is precisely what is being explored in this study.

Dilthey emphasized historicality, which he did not view as a subject of the past but rather as a series of worldviews (Palmer, 1969). *Verstehen* combines individual-psychological and social-historical description and analysis to gain a greater understanding of individuals in their contexts. Dilthey held that individuals could only understand themselves in terms of the lived experiences which spring up out of their depths (Palmer, 1969). For Lijadi and Van Schalkwyk, “remembering and telling stories are necessary tools for the development of a sense of self and identity” (Lijadi and Van Schalkwyk, 2014, p. 4). Indeed, the CLET technique used in this study enables participants to take some time to reflect on the past and “integrate this with the present in view of an anticipated future” (Lijadi and Van Schalkwyk, 2014, p. 4). This can also be viewed in terms of Thirdness in relation to the methodology and the conceptualization of TCKs, with the past as the Firstness, the present as the Secondness, and the anticipated future as the Thirdness. However whether the present as Secondness represents the second culture or the third culture, or the anticipated future represents the first, second or third culture, highlights once again the transient and intangible nature of the third culture and indeed the Thirdness as a mediator.

For Dilthey, “our reflective understanding of life and history must remain determinate-indeterminate” (Stanford Encyclopedia of Philosophy, n.d.). In this sense, there is no wish to generalize the experiences of participants in the context of this paper. Stynes et al. highlight the need for naturalistic research which can propose “an understanding of something with deeper meaning, something that is somehow intangible and personal” (Stynes et al., 2018, p. 160). The grounded theory approach taken in the analysis of the data is also linked with this theoretical lens, and will be clarified further below.

## Data collection process

Two separate workshops were held in enclosed spaces within the university where both authors worked at that time to collect the data. The procedure was explained to the participants prior to commencing, and participants provided their informed consent for data collected to be published by signing an informed consent form which was approved by the Zayed University Research Ethics Committee (ZU18_002_F).

Participants were guided to create a collage based on images provided by the authors from a range of local magazines available in the UAE. They were asked to select images which represent something important or memorable about language and identity in their families’ lives. They were encouraged to talk about the images and the collage in an open-ended manner with the co-investigators and research assistants, both during the process and once the collage was complete. In collaboration with the researchers and research assistants, each individual created an image of relevant experiences and stories about her family’s world (Lijadi and van Schalkwyk, 2014). Each of the two workshops lasted approximately 90 min. The steps recommended by Lijadi and Van Schalkwyk (2014) include:

1. collage making
2. storytelling
3. positioning of the self
4. juxtaposition
5. reflection

The current study placed more of an emphasis on steps 1, 2 and 5—collage making, storytelling and reflection.

For Step 1, the making of a collage, participants were encouraged to select as many images as they could find to describe their experiences. They were asked to arrange these images as they wished and paste them onto the A2 or A3 project board provided.

For Step 2, story-telling, participants engaged actively in life-story remembering (McAdams, 2001) which led to autobiographical memories being generated. They were asked to tell a story about each image on the collage, describe what each image meant to them, and how it contributed to their family’s experience. They were also asked to give reasons for selecting it, the connotations it has for their lives, and the associated thoughts, feelings and meanings each image brings out (Lijadi and van Schalkwyk, 2014).

They were then asked to engage in self-reflection as Step 3. Each participant reflected upon the process of making the collage and writing her or his life story, and summarized their collage. It provided a space in which she could create a sense of coherence among the many I-positions occupied in the process of creating autobiographical memory (Lijadi and van Schalkwyk, 2014).

We have adapted this technique as it gives participants a chance to visualize, display and articulate what different images mean to them and their lives in a relatable and enjoyable manner. While this technique is time-intensive, it allows for participants to take time out to reflect on their experiences in a novel way.

### Participants

Participants were recruited through a Campus Announcement emailed to all faculty, students and staff at the university where both authors worked at the time. Participants were also recruited through the their social circle. Inclusion criteria were as follows:

- living in the UAE with a valid residence visa for a minimum of 2 years
- living with their family (at least one child of school-going age)

Eight participants were recruited, as outlined in Table 1.

**Table 1:** Details of participants.

Nationality	Collage	Passport country/countries	Previous countries lived in	Ethnicity	Time in UAE	Children	In current employment in UAE
Jane	Figure 1	Australia	Lebanon, Australia	Lebanese	10 years	3	Yes
Kara	Figure 2	United Kingdom	UK	British	5 years	2	No
Hilda	Figure 3	New Zealand	Bahrain, Lebanon, Australia	Samoan	15 years	3	No
Vera	Figure 4	Spain	Spain, UK	Spanish	19 years	2	No
Mary	Figure 5	America	America, Bahrain, Kuwait, Pakistan	Pakistani	12 years	2	No
Alison	Figure 6	New Zealand	England, US, Australia, Singapore, Indonesia	New Zealand	3 years	3	No
Sam	Figure 7	United Kingdom	U.K., Canada	British	7 years	1	Yes
Mandy	Figure 8	Canadian	Pakistan, Canada, USA	Pakistani	9 years	2	No

### Analysis

Recordings of the workshops were transcribed by a research assistant and checked by the researchers. All transcripts and images of collages were imported into NVivo 11 qualitative software. The researchers used emergent coding to organize data into themes, using grounded theory as an inductive methodology in order to engage in qualitative content analysis (Cho and Lee, 2014). Employing a grounded theory methodology allows for the discovery of theory from data because it is an open-ended and iterative approach to data analysis. This type of inductive methodology is appropriate for a topic such as this, because prior knowledge regarding the phenomenon (transnational families) in this context (the UAE) is limited or fragmented (Elo and Kyngäs, 2008). Narratives were linked with aspects of the collage images within NVivo, thereby allowing participants’ interpretations of their selection of certain images to be accounted for in the analysis. Nine main themes were initially coded in relation to participants’ lived experiences within transnational families in the UAE. Following on from this, the data were recoded according to the themes presented below. While it was challenging to analyze both textual and visual data in terms of the practicality of cross-checking both aspects against each other, the visual data added an extra layer of richness to the analysis, and helped the researchers to confirm what was represented in the text and vice versa.

## Findings

Nine themes were found emerging from the data set, with the vast majority of these comments being positive. These are as follows: five-star living/ emotive words toward the UAE; the family unit; food; family in home country; religion; language; school; job (self/spouse). There were minimal reference made to the last six themes, therefore the top three themes of five star living, the family unit, and food, will be presented below. The most pertinent findings are supported by verbatim quotations from the participants who created collages for this study.

### Five-star living

All the participants gave their own interpretations of what they believed to be advantages of living in the UAE. This theme emerged particularly through the images the participants selected as well as the verbal explanation.

**Figure 1:**
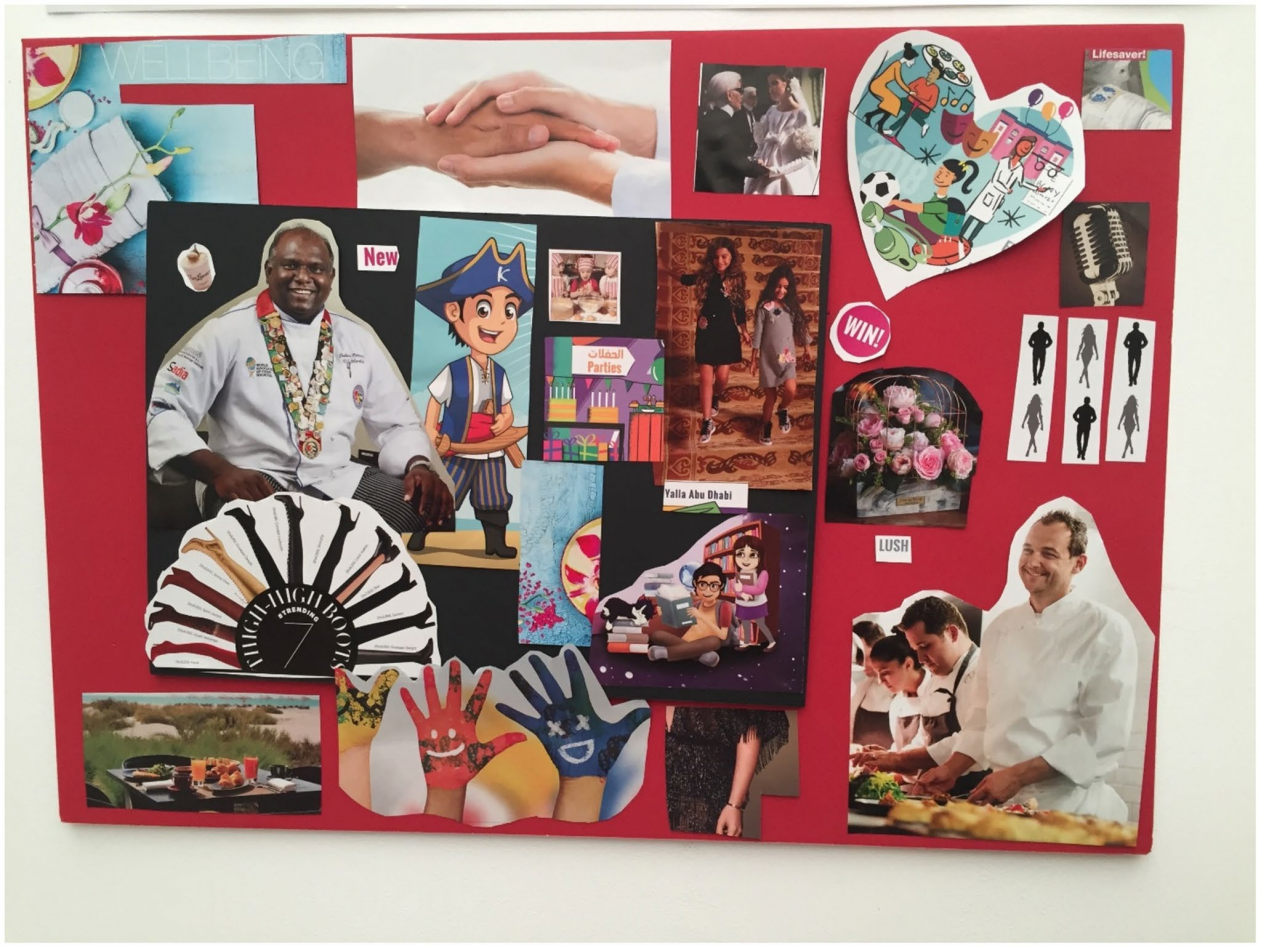
Jane’s collage.

**Figure 2:**
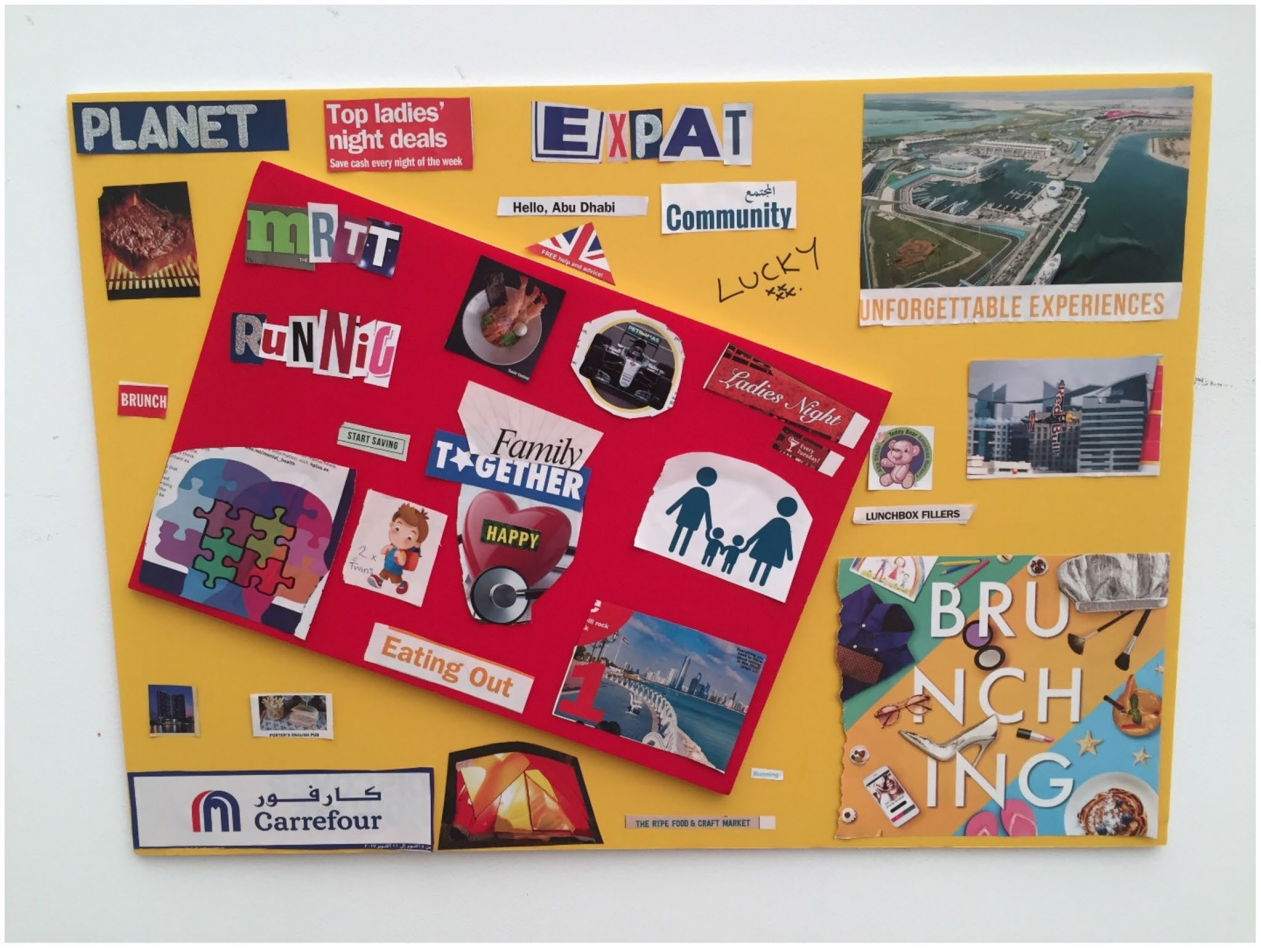
Kara’s collage.

Jane (Figure 1) referred to Abu Dhabi as “five star living 24 h all year round” when discussing her chosen images of Karl Lagerfeld, the word “parties” and a table displaying food. This was similar to what Kara (Figure 2) mentioned. She vividly described the Formula 1 and the Red Bull race as “unforgettable experiences” and also included this phrase in her chosen images as well. Another phrase noted in her images and within her discussion was “lucky”. She emphatically spoke about being lucky to brunch and live in the UAE.

**Figure 3:**
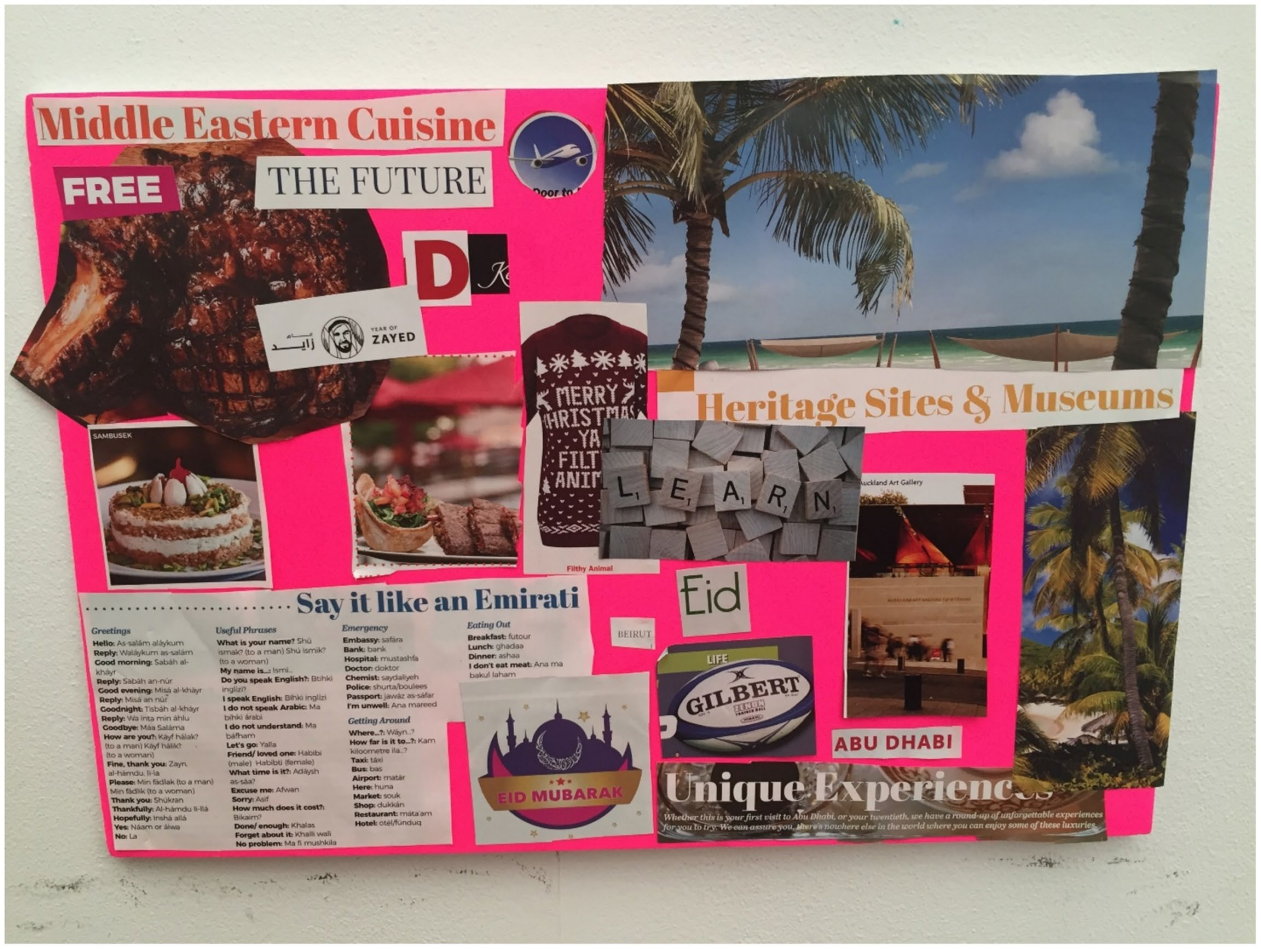
Hilda’s collage.

**Figure 4:**
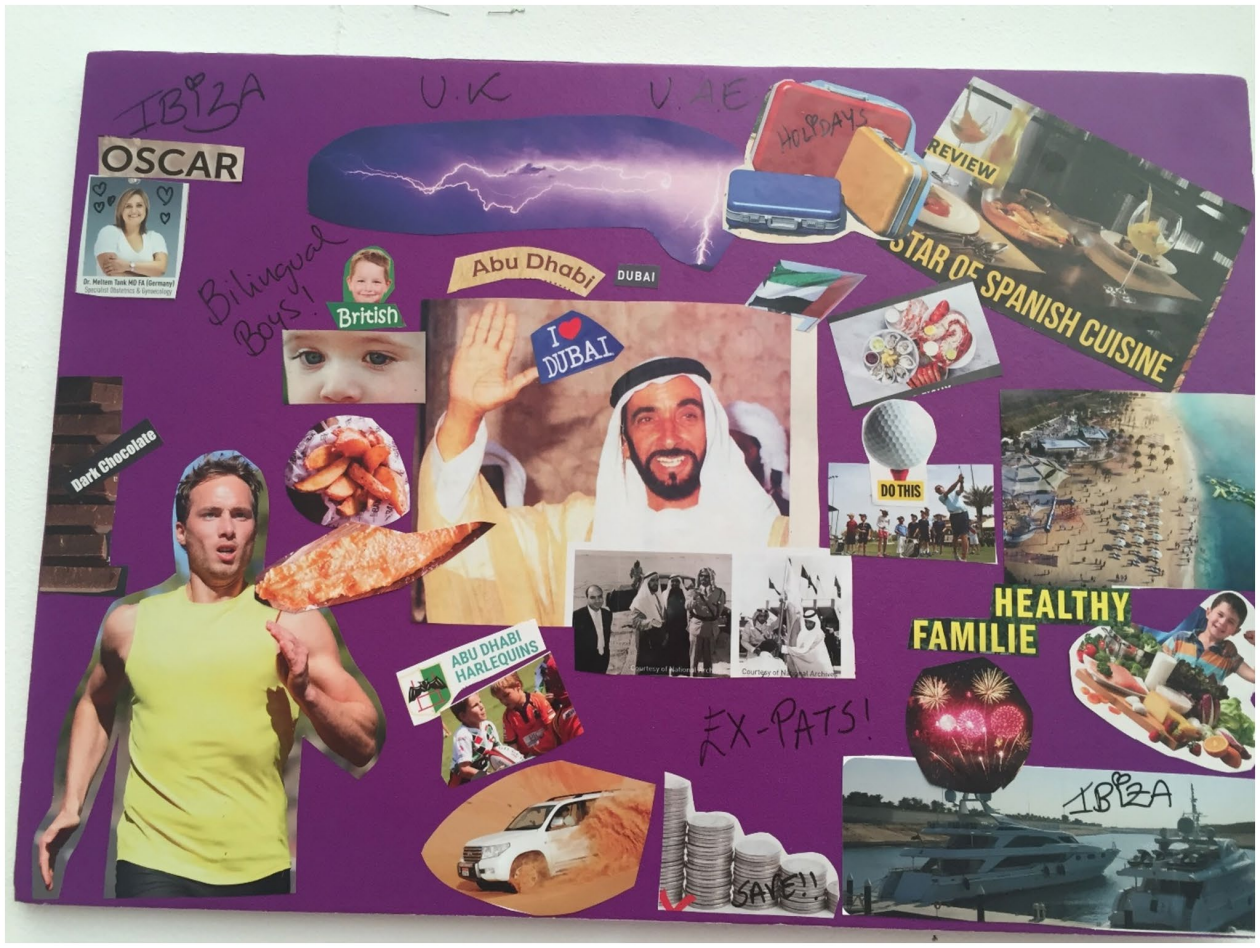
Vera’s collage.

This ethos was also reiterated by Hilda (Figure 3) who chose images of an airplane, and the phrases “free” and “unforgettable experience.” Her explanation to these chosen images were “like the contract has got a lot of perks, I feel like it’s for free, coz when we go back to New Zealand or even Samoa we would never get something like this, you know what I mean.” This statement was in reference to the employment packages offered to expats in the UAE which often include housing and school allowances. Alison and Sam in their discussion while completing the collage also referred to the high level of education available to their children in the UAE, for example “The quality of education is very high.” Vera also agreed with this and mentioned “Now my children are growing up in fantastic private education, they have private healthcare” and the images she chose to include on her collage (Figure 4) were of an aerial view of the beach and a jeep in the desert, indicating the variety of experiences available to her family.

**Figure 5:**
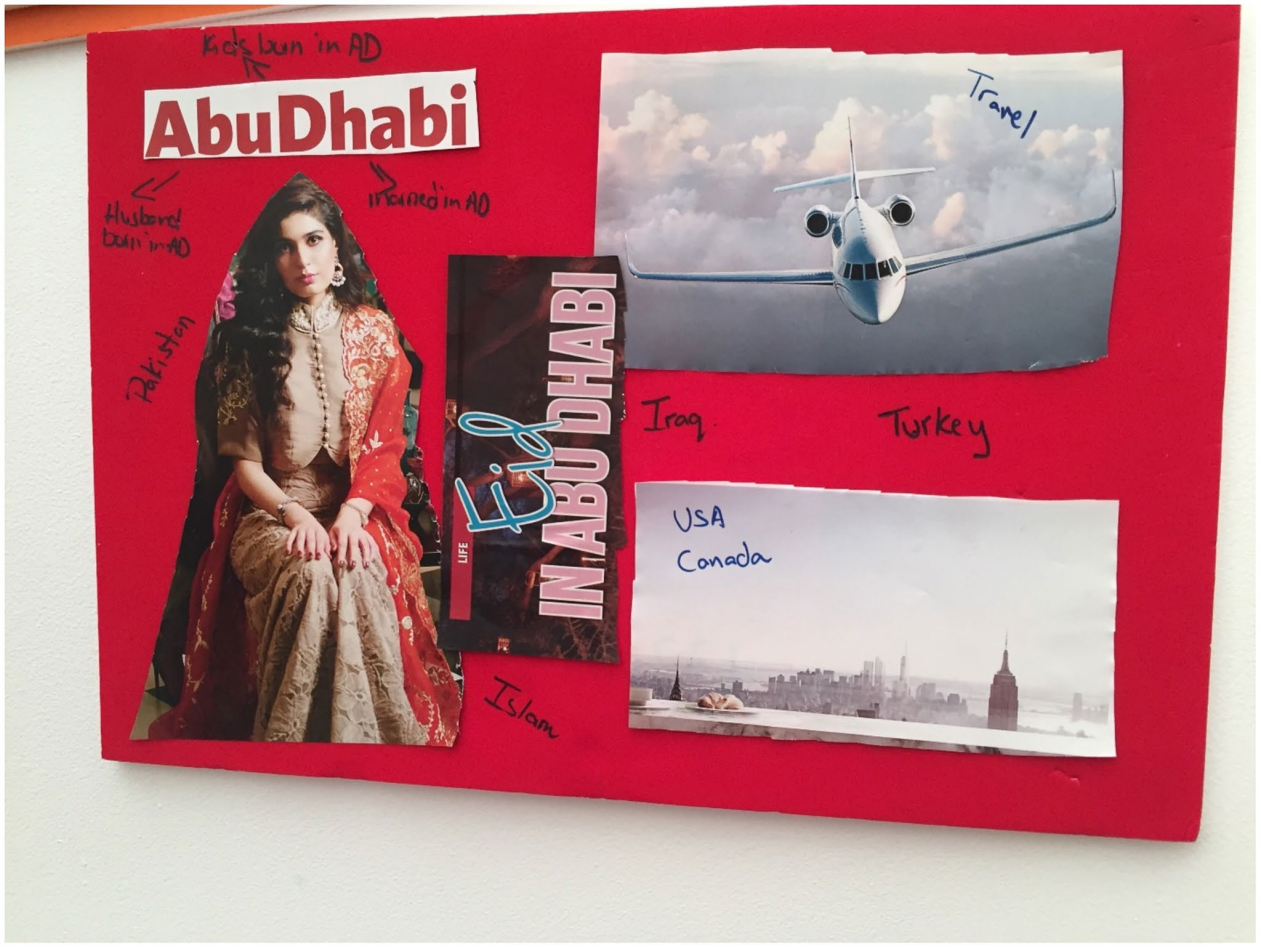
Mary’s collage.

Hilda’s image choice of an airplane was also in reference to their ability to travel and in her words “let us take advantage,” in order to ensure they maximize all the opportunities afforded to them in the UAE. Mary (Figure 5) also chose an airplane to reflect her experience of travel in the UAE and in explanation of her collage stated, “Sparkle and glitter I want to put that in, that’s what UAE is all about.” Kara corroborated this with her explanation as well and identified the UAE as “it’s all about the glitz and glamor.”

All participants demonstrated in their pictures and quotes how living in the UAE was a very privileged life and they were all aiming to take advantage of it. Throughout the workshop, there were also a variety of references to how “lucky” people were to live in the UAE. With regards to the actual collages a number of images and words were cut out and added to collages which were images of the UAE that represented how lucky each participant felt.

### The family unit

The second most popular theme emerging from the data the family unit. Each participant chose images and explained what the images meant to them in respect to their families.

Jane chose four images to denote her family unit-one was a love heart with animation images in it and an image of a person’s hand being held by someone else. She described these images as being “…a representation of our family in Abu Dhabi. It starts with love; love is the essence of everything. Lot of hand holding, lots of cuddles and kisses, and big hand, small hands, dirty hand, you name it.” She also selected an image of two young girls to symbolize her two daughters and also an animated image of two children reading which she mentioned “…there’s a lot of reading as well, there’s a lot of focus on reading, learning and growing.” and she then tied the hand holding image to this photo as well, to ensure she explained all her images signified her family “…and developing and holding hands and taking care of one another as well. All the sisters taking care of one another.”

Kara chose four pictures to demonstrate her family status within the UAE. She cut out letters to spell “EXPAT.” She also stuck the terms “Abu Dhabi” and “Community,” underneath expat. She selected the words “Family” and “Together” over the photo of a heart that had “happy” written in it. Her other two selected images were of an animated image depicting a family of four (male and female holding hands with two children) and an image of a jigsaw puzzle which consisted of four pieces… “when we came to Abu Dhabi, me, my husband, and we have got twins and the rule was we needed to be happy, if one wasn’t happy, then we leave. So this is what the “Family together” and the “heart” and “happy” represents, the two little boys here and then this picture here… And a picture of jigsaw and 2 minds, this is our family who are together, as simple as that.”

The family unit was also seen in the collage created by Mary. She wrote “Husband born in AD” and also wrote the words “Iraq” and “Turkey” on her collage. Mary spoke of her husband in relation to the words she had chosen to write “…my husband was born here, my in-laws still live here, and my kids were born here… Because my husband is actually an ethnic Turk from northern Iraq, so when the first gulf war happened, they did not want to have the Iraqi passport, so they went to Canada.” She selected an image of the skyline in NYC and wrote “NYC” and “Canada” on the image. She explained “I put also United States and Canada, because I was born in the states and my husband lived there as well. My kids are American and Canadian, but they have been to Canada only once, but since my family lives in the states, that’s actually the only place we travel to. So I’ve put this as North America even though it’s New York.”

**Figure 6:**
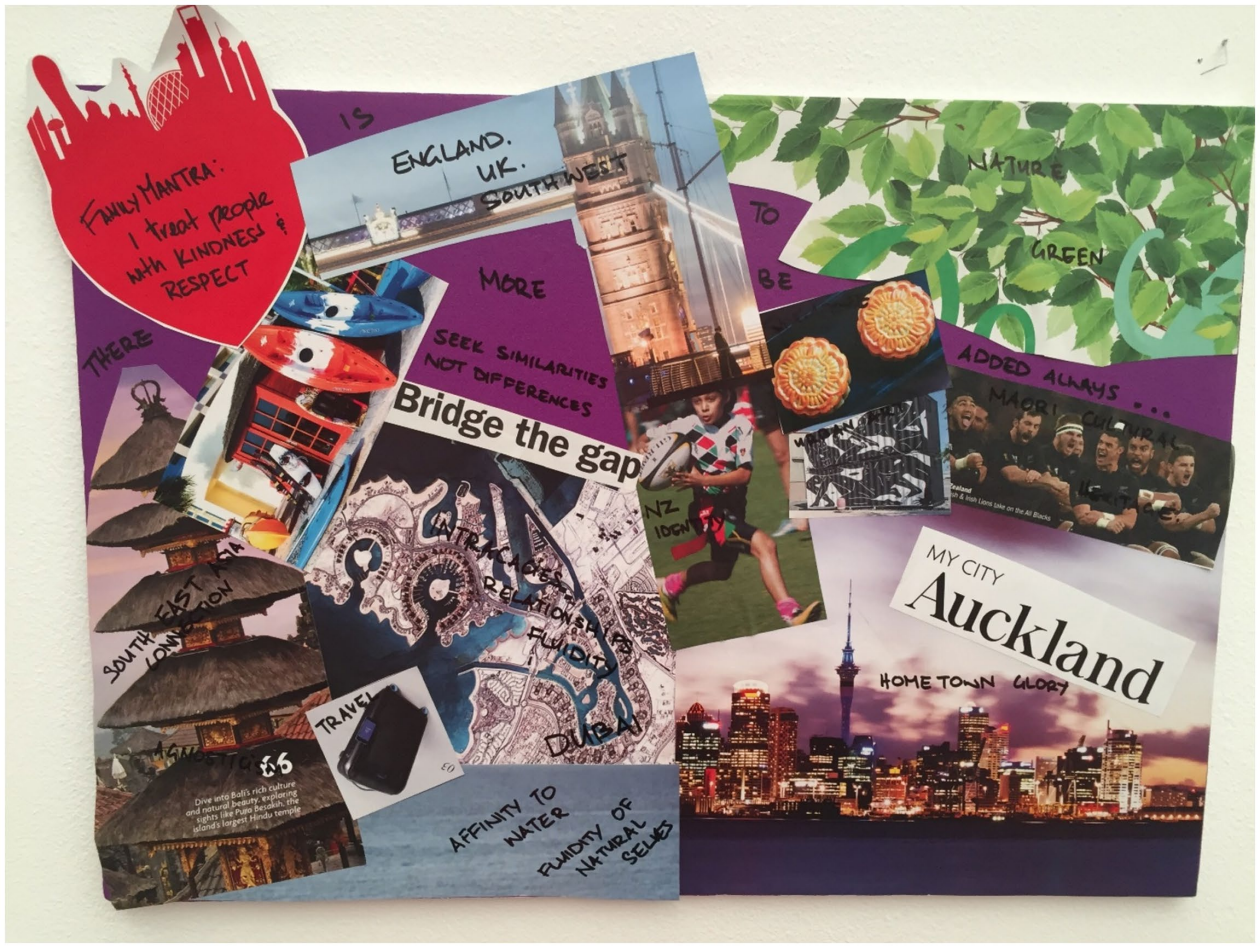
Alison’s collage.

Both Alison and Sam also spoke of their husbands and how the images they had chosen in their collages represented their husbands and families. The originating countries of their families was an important topic of conversation while completing their collages. Alison (Figure 6) noted “…I’m from New Zealand but I was born in Singapore, lived in Singapore, but my husband was born in England, but he grew up between Singapore and Dubai and we are back here and we have been here for 2 years now.” Sam spoke of both her husband and her son “…my son, Thomas, he’s half Canadian and half British… he was born in Canada. But we have been here for about 5 years, so he’s been here since he was a baby, and that’s one of the major reasons we do not want to leave because of the school….”

Hilda similarly spoke of her family’s origins while completing the collage, explaining why she had chosen the word Beirut to be included in her collage “…here’s Beirut, coz he’s from Beirut, so the kids are Samoan and Lebanese… fees like even within us, we own quite different passports, different culture, language, so it’s always good to explore as well.”

### Food

Another primary theme to emerge from the data was food. The majority of the participants included food images and discussions in their collages and noted how meaningful food was within their cultures and their families.

Vera included four images in her collage which depicted this theme. These images were of a set table with the words “Spanish Cuisine,” an image of dark chocolate, an image of a young smiling boy used to symbolize her son and an image of salmon and chips. She explained “…plates of seafood and Spanish cuisine, lots of food, I miss the food from home… in the mainland, it’s more meat related and a lot of game and a lot of hunting meats, pigeons, rabbits… But in the island or the coastal area, there is a lot of seafood, fish and seafood.”

**Figure 7:**
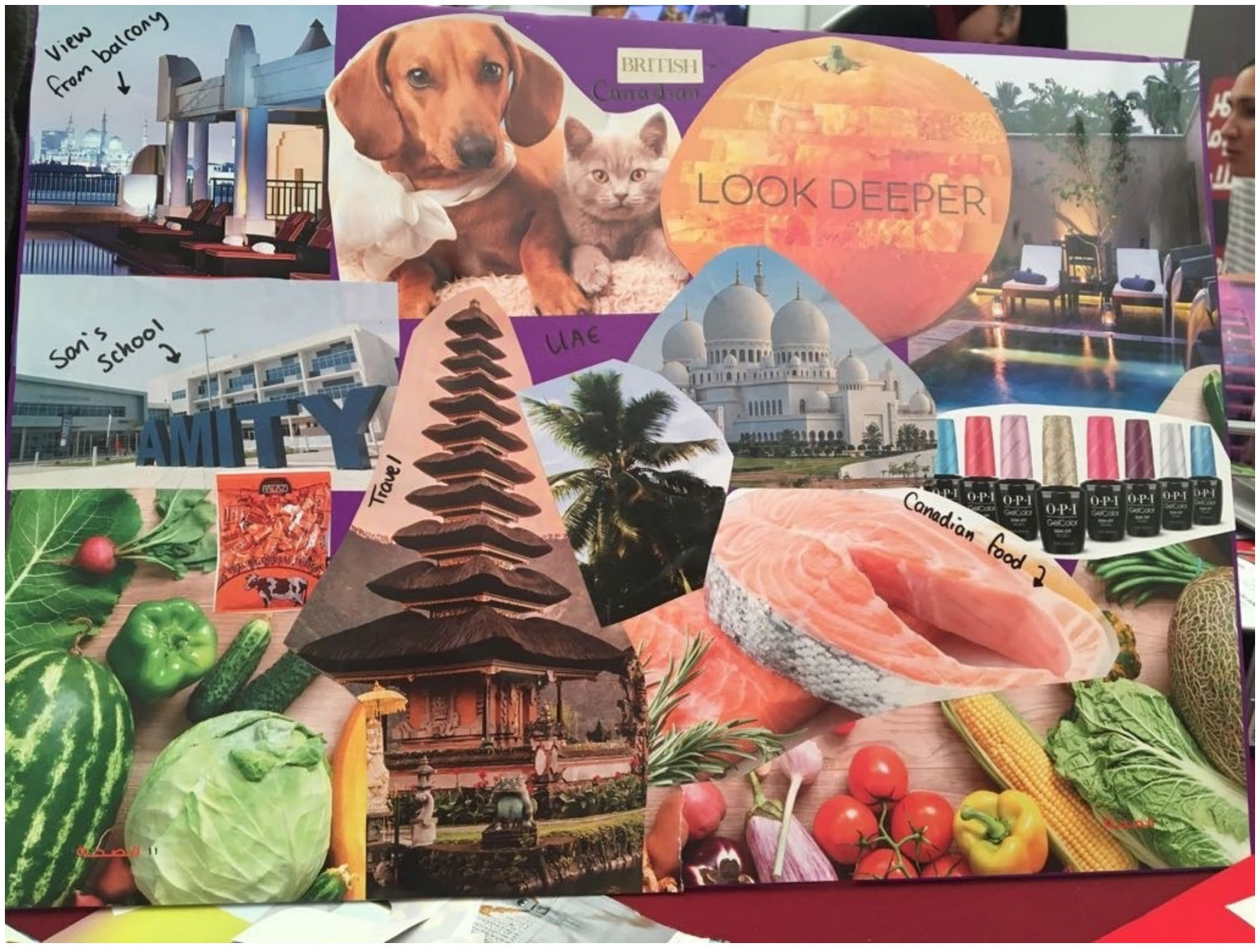
Sam’s collage.

Sam (Figure 7) also included images of food—one image showing vegetables and one showing fresh salmon. She wrote “Canadian Food” over the image of salmon and explained “This represents Canada. So, my husband is Canadian, and we lived there for 10 years and the salmon over there is amazing, fresh salmon… Yeah but the other thing you see is that my son, he is 6 years old and he loves vegetables, we are very lucky because his favorite thing is going to farms and picking vegetables and he knows the names of each vegetable and I guess this is a part of his identity so that’s good.”

Kara’s collage comprised of food images and words relating to food. She chose two images displaying food—one of a steak and another of prawns. In addition to this there was a photo of sandwich and chips with “Porters English Pub.” She also chose to insert the words “Brunch,” and “The Ripe and Food Market.” Her explanation in relation to these images was “…There’s a lot of food pictures, there’s a lot of pictures which say free ladies’ nights, which is an amazing thing to try to explain to somebody that we have the option to go to a ladies night and you can drink and eat for free, it’s an amazing thing we have got… there’s a picture of Porter’s English pub, because I love the pub back in the UK, it’s just the food is good, I love pub food.”

Hilda was particularly emotive toward food within her family and culture: “I’ve got food here, because it’s like culture, because family time is always around food, definitely in the Lebanese culture as well. And I was telling Mandy (other participant), that when we have my in-laws come over, like they start in the morning like 8 o’clock and they start preparing dinner, the food is amazing but it’s a lot of preparation and things like that. Even when I’m home my mom cooks Samoan food, she lives in New Zealand, it takes forever to make but you know I do not do that. So, like Samoan culture and Lebanese culture is always around food.” Her accompanying images with this were images of different food cuisines and the words “Middle Eastern Cuisine.”

Jane also spoke about food with emotion and explained her included food images with “…We do a lot of outdoor cooking and food is a big part of our lives as well and our culture and also the joint cooking that we get ourselves involved in.” Her images depicted an al fresco dining setting and chefs preparing food.

**Figure 8:**
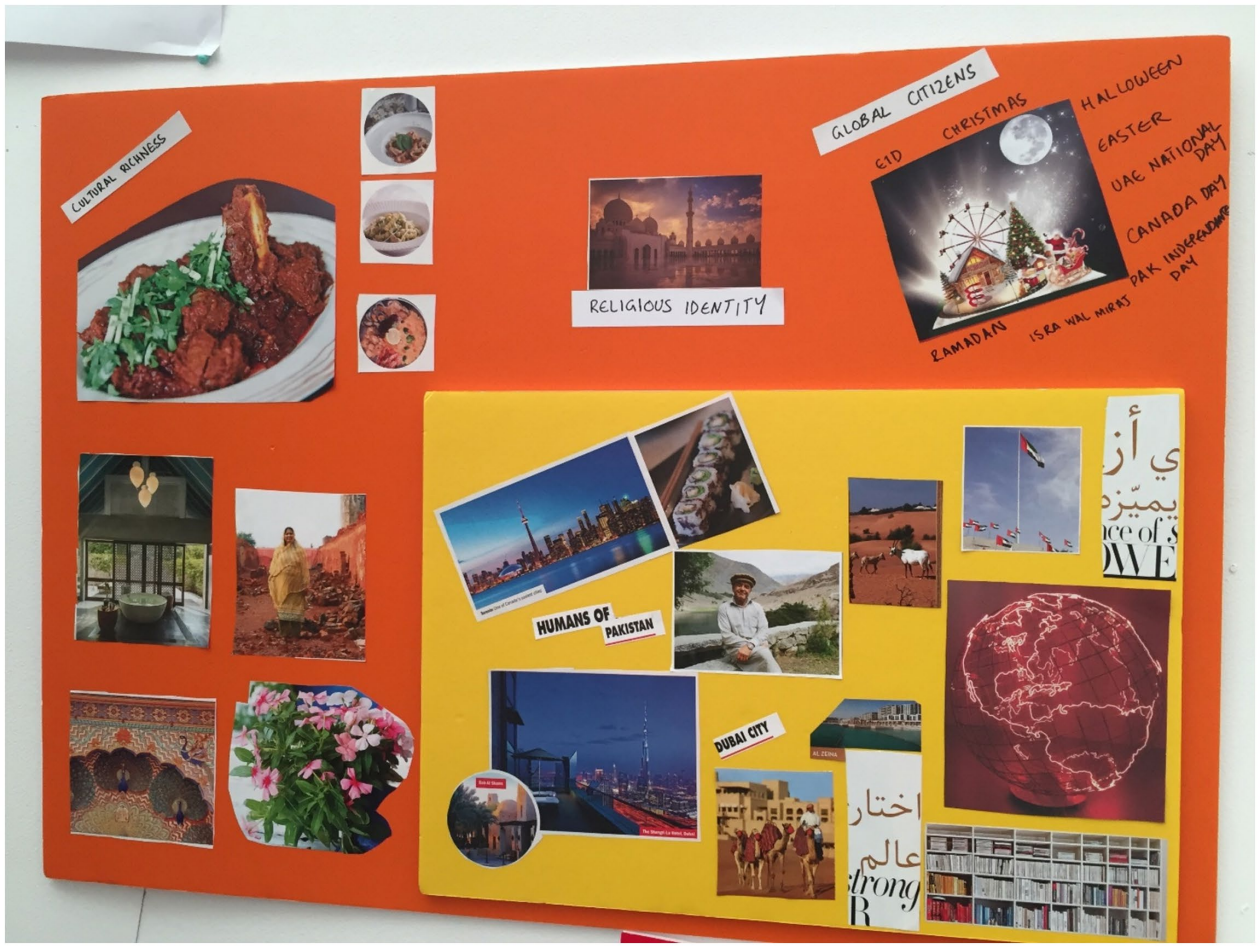
Mandy’s collage.

Mandy (Figure 8) noted food to be an important factor in her culture and family too. She included 5 different images displaying different cuisines and stated “…My house always has detailed discussions about food… And there’s food because our culture is all about food.”

## Discussion

It is clear from the images chosen and examples given that while all participants made multiple references to how lucky they were to be living in the UAE, their home culture was very important to them. This love of the home culture was often expressed through references to food, and how food brings the family together, whether that is the family in the home country, or the family in the host country. Most participants referred mainly to the food of their home country, but did make references to all the different foods that they have experienced in the UAE. The other two main themes represented were five-star living and the family unit.

In using *Verstehen* as a theoretical lens, the experiences narrated and visually constructed by these mothers reveal the triadic nature of transnational families. Firstness is represented here by food, secondness as five star living, with thirdness represented by the family unit. The family unit is something that is very important in the narratives of these third culture mothers. The dialogical selves were apparent throughout the narrative. All of the versions of “me” were consistently navigated by references to the family unit as the glue that brings them together, with the family unit offering the link between the host culture and the home culture, and a springboard for exploring other cultures. This echoes Bhabha’s in-between space, and indeed in this sense the family can be seen as a safe space to explore the cultural past and present, and to negotiate cultural innovation. Similarly, de Waal & Born’s study (de Waal and Born, 2021) found TCKs to define their sense of belonging in terms of their personal relationships, rather than geographical locations. The representation of the family space as Thirdness demonstrates Raggatt’s dialogical multiplicity in that this study presents mothers who constantly shift between family memories and current family life. They draw on family experiences to connect the separate stories of various “I”- positions. How different families do that varies widely across cultures and depends greatly on the family’s relationship between the host culture, home culture, and interstitial culture.

The triadic nature of the TCI/ TCK/ transnational family is well represented by these phenomenological categories of Firstness, Secondness and Thirdness. Firstness seems closer to country-of-origin culture, while Secondness seems closer to host country culture. Thirdness is still difficult to quantify although it seems apparent that the dialogical self, continually evolving and shifting from one position to another, can find meaning through the family unit. Similarly to the way in which TCKs relate to each other within the interstitial space, where they tend to relate more deeply to other TCKs, the members of the expatriate family relate more deeply to each other. The expatriate family unit as an entity gives a meaning to thirdness, which remains difficult to define. However, the family unit has an identity in and of itself, as a transcultural space. Interestingly, the theme of “food,” so closely linked with family life, brought to mind the so-called “invention” of Emirati cuisine, as explored by Martín (2021). Martín discusses this invention as a representation of nation-building in the UAE, where Emirati food incorporates traditional, modern and fusion foods, and argues that it highlights the connections between nation-building, social transformation and food. In this way, even the development of UAE cuisine can be seen as triadic, and negotiated in ways similar to the experience of transnational families.

Participants felt free to express themselves given the structure of the CLET, which offered a unique way to interpret their lives as transnational families, holding to the concept of the “life-context” of *Verstehen*. As an open-ended study, using the CLET to assist mothers to explore their lives as expatriates within a rentier state proved successful and also as something which was beneficial to them to give them a chance to reflect on their own lives.

It should be noted that the expatriates interviewed for this study were all living under similarly middle-class socioeconomic circumstances and fell under the umbrella of the traditionally defined TCK (Pollock and van Reken, 2017), similarly to the “Disneyland” narrative referred to by Tanu (2016) and the exotic sense of the expatriate lifestyle mentioned by Korpela (2016). For context, Table 1 outlines the varying backgrounds of each mother, in terms of number of countries lived in, length of time and ethnicity, with some mothers working outside the home, some earning the main source of income within the household, and some as trailing spouses. Despite these differences, the socioeconomic context remains similar for all participants. This study did not take into account the broader terms referring to TCKs mentioned by Dillon and Ali (2019), and therefore is not representative of all types of transnational families in the UAE. There are many different socioeconomic strata in the UAE and each family’s experience of the expatriate lifestyle will be different because of this. This is a limitation of the current study, and it would be of benefit to the field to include individuals similar to those included in Gallagher’s study (Gallagher, 2024).

## Recommendations

It is apparent that maintaining a relationship between the expatriate family and the home culture appears to be an important part of life for transnational families in this context. This study will have implications for how these families operationalize their third culture experiences in the context of multilingual identities, which is a theme that did not appear here but would be worth specifically exploring. Since family is one of the only constant relationships for TCKs, the success of transitions between places will depend on how parents and children anticipate, negotiate and co-construct these identities within families as well as in the larger sociocultural environment. The use of CLET as a technique will provide a new lens of using visual research methods in a range of contexts and with different populations across the Middle East and wider in the Asia-Pacific region.

It should be noted that this study took place prior to the COVID19 pandemic, which has had huge implications for families all over the world, including expatriate families. Said et al. (2021) found that mothers in the UAE were experiencing many challenges in playing multiple extra roles in their children’s lives—in addition to the usual caregiving, they had to take on additional responsibilities in their children’s education. The role of mothers in transnational families is an area for further exploration. Furthermore, the implications for TCKs in the context of travel restrictions to their home countries will be interesting to explore, in particular the challenges of maintaining the close relationship between the home culture and the host culture, and the intervening space we know as the third culture. More recent work by Hopkyns (2023) and Baycar (2023) highlight a new form of “banal” nationalism among expatriates in global cities such as Dubai and Abu Dhabi. This type of nationalism promotes “unity through diversity,” which speaks to a new national identity that includes expatriates rather than excluding them, despite a lack of permanent residency. The transnational outlook articulated by participants in Errichiello & Nyhagen’s study (Errichiello and Nyhagen (2021)), and the ambivalent identities presented by Khonder (2022) as “a source of strength in a highly globalized and multivalent society” (Hopkyns and Zoghbor, 2022, p. 9) warrant deeper exploration in other global cities, potentially using technique similar to the CLET. Narratives of transnational families are likely to continue evolving in dialogical multiplicity in these fast-paced societies, and it will be interesting to explore the role of family as an in-between space in navigating these multiple “I” positions over time.

## Data Availability

The raw data supporting the conclusions of this article will be made available by the authors, without undue reservation.
